# Retraction Note: Role of the gut microbiome in chronic diseases: a narrative review

**DOI:** 10.1038/s41430-024-01467-z

**Published:** 2024-06-26

**Authors:** Amrita Vijay, Ana M. Valdes

**Affiliations:** 1 Division of Rheumatology, Orthopaedics and Dermatology, School of Medicine, The University of Nottingham, Nottingham, UK; 2 NIHR Nottingham Biomedical Research Centre, Nottingham University Hospitals NHS Trust and University of Nottingham, Nottingham, UK

Retraction to: *European Journal of Clinical Nutrition* 10.1038/s41430-021-00991-6, published online 28 September 2021

The Editor-in-Chief retracted this article because it contains text that substantially overlaps with the following articles, [1–4]. The authors agree with this retraction.
